# Predictive model to design for high cycle fatigue of stainless steels produced by metal additive manufacturing

**DOI:** 10.1016/j.heliyon.2022.e11473

**Published:** 2022-11-10

**Authors:** Ahmad Serjouei, Shukri Afazov

**Affiliations:** Department of Engineering, School of Science and Technology, Nottingham Trent University, Nottingham, NG11 8NS, United Kingdom

**Keywords:** Fatigue life, Additive manufacturing, Defects, Surface factor, Defect factor

## Abstract

This work develops a predictive model of S–N curves capable of proving the lower and upper bounds for fatigue behaviour of parts fabricated using different metal laser-based additive manufacturing (AM) and post-processing technologies. Alongside the ultimate strength of the material and its endurance limit, a surface factor and a defect factor are incorporated in the model to consider the effects of the AM process induced defects on the S–N curves. The model is correlated to comprehensive load-controlled fatigue experimental data from the literature for 316L stainless steel (SS) samples manufactured using metal laser-based AM technologies of various process and post-process conditions. It was demonstrated that the proposed model can capture and represent the effects of the induced defects as well as the mean stress effect. The value of the proposed model is that it can be integrated into existing industrial design workflows for fatigue assessment of AM 316L SS. Furthermore, it has the potential to be extended to other AM materials.

## 1 Introduction

Additive manufacturing (AM) has been recognized over the last three decades as one of the revolutionary manufacturing technologies in various industries such as aerospace, healthcare and automotive. Metal AM represents different laser powder bed fusion (LPBF) technologies such as selective laser melting (SLM), selective laser sintering (SLS), direct metal laser sintering (DMLS) and electron beam melting (EBM).

There are numerous AM process variables, e.g., beam parameters [1, 2, 3], scanning parameters (pattern, speed, power) [4, 5, 6, 7, 8, 9, 10], powder characteristics and distribution [11, 12, 13], which can induce internal defects and largely affect the surface roughness. For example, Yang et al. [1] showed that balling, un-overlapped defects, large re-heated zone, and large sub-grain size occur due to use of a large laser spot with low volumetric energy density in LPBF manufacturing of AISI 420 stainless steel (SS) parts. Whip et al. [4] showed decrease of surface roughness parameters, namely average roughness, and maximum valley depth, reduced dale height and reduced valley depth, by increasing the laser power used for fabrication of nickel-based superalloy 718 using LPBF technology. Read et al. [9] showed significant influence of laser power, scan speed, and interaction between the scan speed and scan spacing in LPBF on the porosity development of AlSi10Mg parts. Baitimerov et al. [12] used three different batches of gas atomized AlSi12 alloy powders from different manufacturers with different particle size and distribution, morphology and chemical composition to fabricate LPBF samples. They showed that both the flowability and powder layer density significantly affect the LPBF processability of the AlSi12 powder.

To improve the surface and material performance, different post-processing methods have been employed in AM, which are classified mainly into two forms (1) surface treatment methods (e.g., machining, shot-peening, laser shock peening, laser polishing): to improve the surface quality; and (2) heat-treatment methods (e.g., stress relief, precipitation hardening, annealing, hot isostatic pressing - HIP) to reduce the residual stress and porosity as well as to modify the material microstructure and properties.

There are abundant literature research focusing on strain- or load-controlled fatigue behaviour of AM parts fabricated using various process and post-process conditions. It has been suggested that five parameters are key to describe the influence of a defect on the fatigue limit of a metallic material: size, type, position, morphology and loading [14].

To date, the research on the fatigue evaluation of AM parts has been focused mainly on three aspects related to defects consideration [15]: (1) Considering statistical spread of defects through fatigue data statistics irrespective of different defects attributes; (2) Theoretical studies, for example modelling of fatigue crack growth from process induced micro-crack; (3) Extracting the shape of the defects and their distribution in AM parts using computed tomography (CT) to make high-fidelity material models and then study their behaviour under loading using methods such as the finite element. Researchers have utilised CT to accurately find the position, size and morphology of the defects [16, 17]. Recently, some research studies have combined the abovementioned aspects. For example, Romano et al. [18, 19] exploited the combination of fracture mechanics and statistics of extreme for the distribution of defects and their critical size measured through CT for fatigue failure analysis of AM AlSi10Mg parts. Sanaei et al. [20] used 2D digital microscopy and 3D X-ray micro-CT techniques to explore the characteristics of defects such as shape factor (sphericity) and aspect ratio and their distribution in laser powder bed fusion (LPBF) for Ti–6Al–4V and 17–4 P H SS parts with different process and post-process conditions as well as their effects on fatigue properties.

There are research studies that consider the effect of defects on the fatigue behaviour of AM parts. For example, Pegues et al. [21] proposed a simple method to estimate the fatigue endurance limit of AM Ti–6Al–4V parts. In their method, which is based on a previous approach utilized to predict the fatigue limit of steels, surface roughness factors are used to estimate stress concentration effects.

There are limited works that discuss the statistics of fatigue research data for a specific AM material. Solberg and Berto [15] compared the fatigue behaviour of 316L SS and 17–4 P H SS, Inconel 718 and Ti–6Al–4V wrought materials with their AM counterparts manufactured in as-built or post-processed conditions. They emphasized the adverse effects of defects and the positive effects of post-processing on the fatigue behaviour of as-built AM parts. Paul et al. [22] catalogued fatigue data of AM 316L SS parts with different process and post-processing conditions and discussed the common trends observed in their S–N curves. Li et al. [23] investigated the governing fatigue failure and factors that influence the process parameters, defects and microstructure for Ti–6Al–4V parts. They also conducted a comprehensive comparison of parts fabricated by directed energy deposition (DED), EBM and LPBF with their cast and wrought counterparts. In doing so, they normalized the data from different resources using a unifying equation, $\sigma_{eff}=\sigma_{\max}{\left(0.5\left(1-R\right)\right)}^{0.28}$, where $\sigma_{eff}$, the effective stress, is the effective maximum stress value under fatigue loading with stress ratio R=−1 and $\sigma_{\max}$ represents maximum stress in a fatigue loading with stress ratio, R. Beretta and Romano [24] conducted a comprehensive review on experimental axial load-controlled fatigue analyses, with stress ratios R=0.1 and R=−1, of AM Ti–6Al–4V and AlSi10Mg samples as well as their cast and wrought counterparts. They applied a fracture mechanics approach to find the material crack propagation threshold and fatigue limit and showed that their model can predict fatigue trends for both materials.

While the experimental works abound in the literature on load- or strain-controlled fatigue behaviour of AM parts manufactured using specific metal AM technology with certain process and post-process conditions, models providing design boundaries for extreme fatigue loading scenarios is yet to be developed. Despite the present approaches to characterise the physics of crack initiation under cyclic loading, there is still a lack of standards to recommend a pragmatic approach during the design stage against fatigue in AM. Therefore, there is a need to translate the scientific knowledge to a pragmatic engineering approach that can be used in design for AM against fatigue. The novelty of this paper is to utilise an engineering approach to predict S–N curves by incorporating surface and defect factors into the established endurance limit approach. The aim of the paper is to propose an overarching model capable of predicting S–N curves with lower and higher bounds for 316L SS parts fabricated using metal AM technologies undergoing load-controlled fatigue loading. The model adds a new defect factor on the top of a surface factor that already exist in the endurance limit approach. These factors take into account the effects of process and post-process manufacturing variables on the fatigue behaviour of AM 316L SS. The model also considers the mean stress effect using the R ratio which is important for dynamic loads where the mean stress is constantly changing.

## 2 Description of the fatigue life predictive model

### 2.1 Background on fatigue life of conventionally manufactured ductile materials

**Figure 1:**
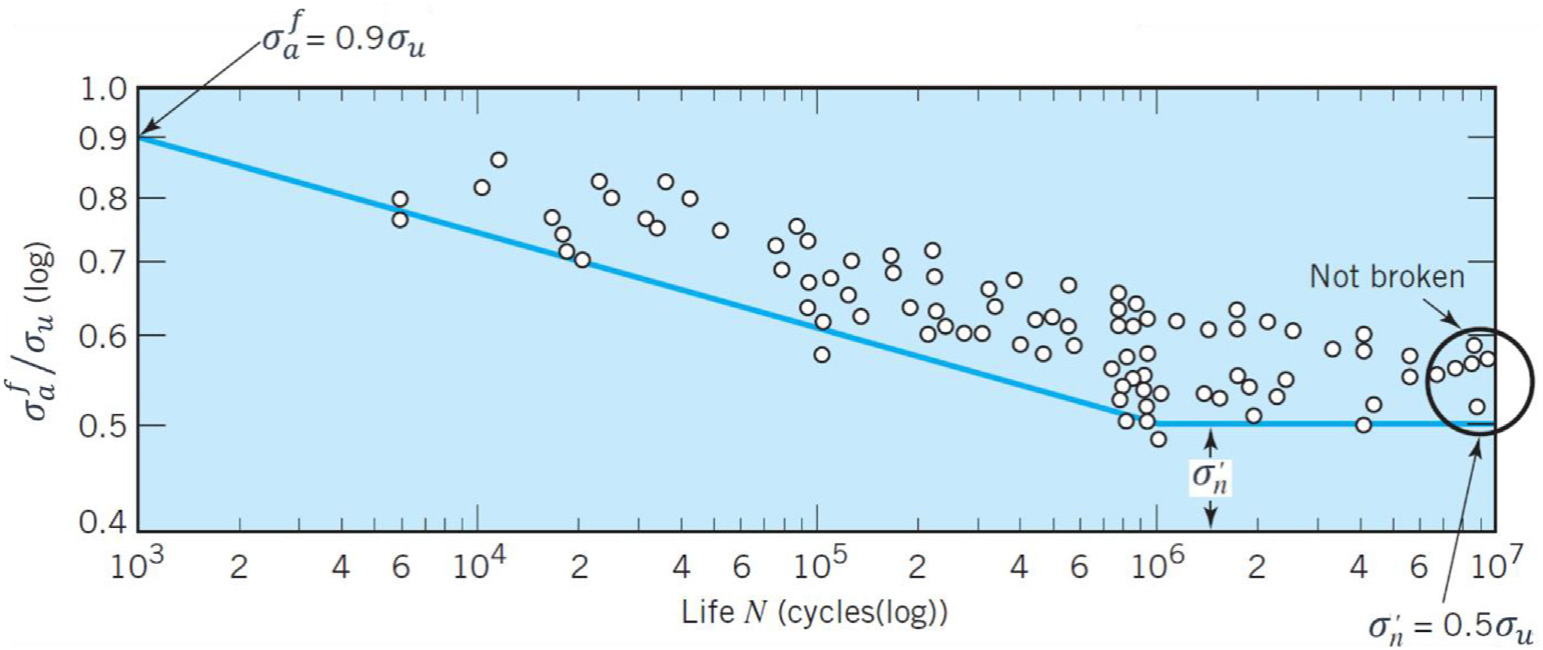
Generalized S–N curve for wrought steel under standard R. R. Moore rotating-bending fatigue loading with superimposed data points; adapted from [25].

*Fatigue strength* for a material, denoted by $\sigma^{f}$, is defined as the intensity or magnitude of the applied stress which results in failure at a specific number of cycles. It is known that a basic *endurance limit* (or long-life endurance strength), denoted by $\sigma_{n}\text{,}$ exists for conventionally manufactured ferrous materials. The endurance limit is defined as the largest value of alternating stress that material can sustain without failure up to an indefinite number of cycles [25]. A “knee” is observed in the S–N curves, in the range of 10^6^-10^7^ cycles, for materials with an obvious endurance limit. A sharp “knee” is observed for nonferrous materials such as the conventionally manufactured aluminium alloys. Figure 1 depicts a generalized S–N curve for wrought steel with superimposed data points using the endurance limit approach in which the relationship between fatigue strength and number of cycles can be found by only knowing the ultimate tensile strength (UTS) of the material and its endurance limit.

The endurance limit for ductile materials can be estimated by applying correction factors to the endurance limit of specimens under rotating-bending fatigue loading [25]:

(1) $$\sigma_{n}=k_{l}{k_{t}k}_{g}k_{s}k_{r}\sigma_{n}^{\prime }$$

where $\sigma_{n}^{\prime }$ refers to the special case of endurance limit for standard R. R. Moore rotating-bending fatigue test under ideal conditions for specimens with carefully polished surfaces, $k_{l}$ is a load factor accounting for bending, axial or torsional type of loading; $k_{t}$ is a temperature factor accounting for the temperature effect on the material; $k_{g}$ is a size factor or a gradient factor which considers the effect of geometry on the fatigue strength; $k_{s}$ is a surface factor considering the effect of surface finish on the fatigue strength; $k_{r}$, is a reliability factor addressing the fact that a lower endurance limit should be used for a more reliable (above 50%) fatigue strength estimation.

### 2.2 Application for endurance limit approach for AM materials

The fatigue life at high cycles $\left(>10^{3}\right)$ can be predicted using the stress-based approach (S–N curves). The endurance limit approach, explained in the previous section, has been used in industry to obtain fatigue stress amplitudes, $\sigma_{a}^{f}$, for different metals by approximating the fatigue stress amplitudes for conventionally manufactured steels at 10^3^ and 10^6^ cycles using [25]:

(2) $$\sigma_{a}^{f}=0.9\sigma_{u}\;at\;10^{3}\;cycles$$

(3) $$\sigma_{a}^{f}=0.5\sigma_{u}\;at\;10^{6}\;cycles$$

where *σ*_*u*_ is the UTS.

At low cycle fatigue (thousand cycles and less), the surface finish effect is generally negligible due to a high degree of plastic deformation experienced by the specimen and crack propagation mechanisms dominates, hence the condition of the surface finish has minimum (negligible) effect on the fatigue life [26]. This is contrary to the domination of crack initiation mechanism at large number of cycles (greater than 10^6^) in which the surface condition highly influences the fatigue strength. Therefore, Eq. (2) is used for the low cycle fatigue in this work for all surface conditions while Eq. (3) is modified to account for the effects of AM at high cycle fatigue. It is well-known that the metal AM technologies introduce defects such as surface roughness, internal porosity and micro-cracks to the AM parts. To apply the same analogy as in Eq. (1) for fatigue of AM materials at 10^6^ cycles, we introduce a surface factor, $k_{s}$, and a defect factor, $k_{d}$, to Eq. (3):

(4) $$\sigma_{a}^{f}=0.5k_{s}k_{d}\sigma_{u}\;at\;10^{6}\;cycles$$

The position of the defect can define the type of defect. For instance, a surface defect is directly located at the surface; a sub-surface defect is an internal defect very close to the surface; and an internal defect is in the bulk of the material without being exposed to a free surface [14].

In our model, the surface factor is solely related to the presence of a defect on the surface while the defect factor is related to internal defects in the bulk of the material. The surface and defect factors are assumed to be intercorrelated, hence both factors are incorporated in the model. There might be cases where the surface can be without defect $\left(k_{s}\approx 1\right)$, while inside of the sample far from the surface can have a defect (defect factor less than 1, i.e., $\left(k_{d}<1\right)$ – i.e., AM parts with polished surfaces. In relation to this, the ASTM E155-15 standard [27], which categorises cast components to defective and non-defective based on the defect shape and size only, can be used as a basis to further characterise defects based on their location as well.

**Figure 2:**
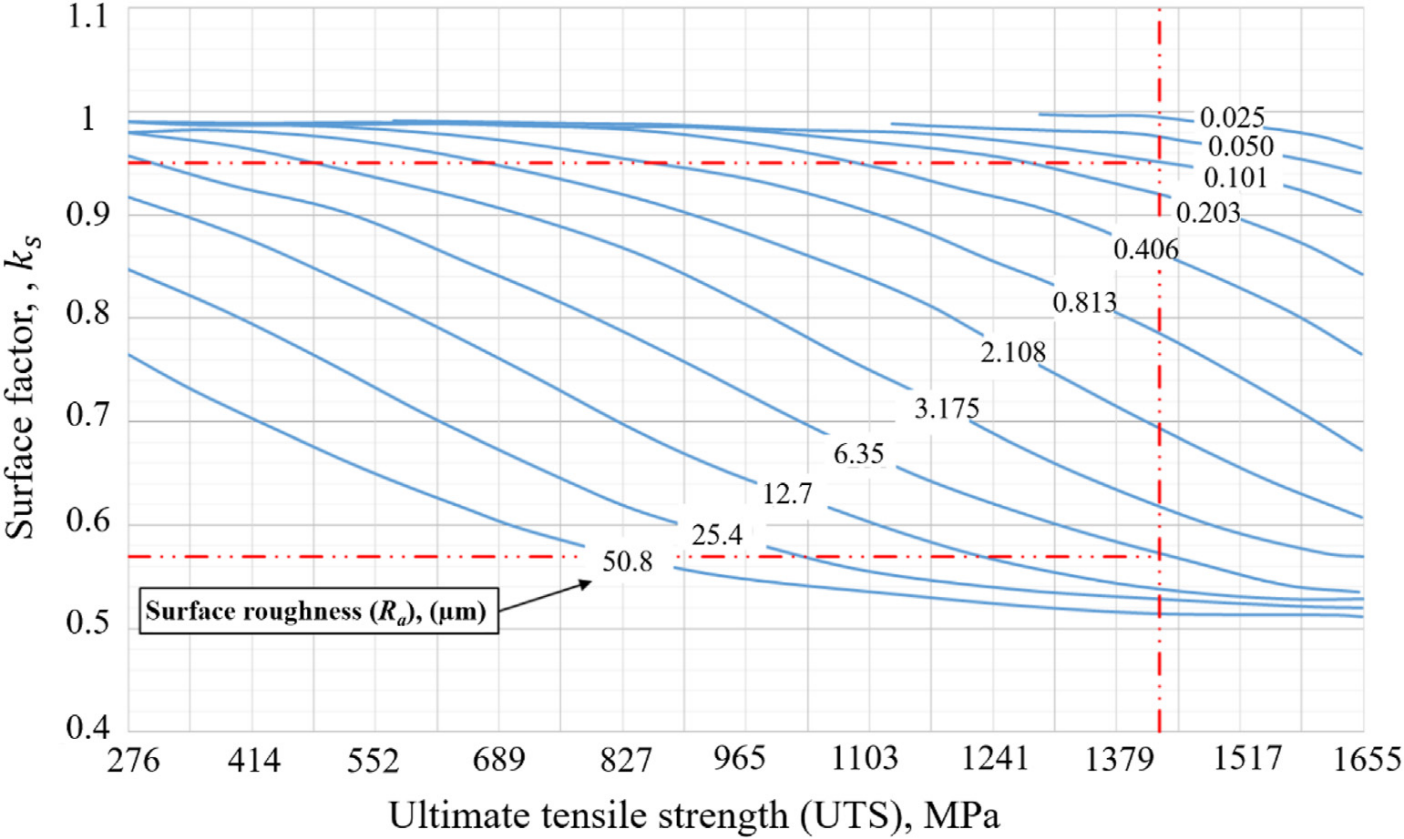
Variation of the fatigue surface factor for metal parts plotted against UTS and surface roughness $\left(R_{a}\right)$ of the material; modified from [26]. Note that units for UTS and $R_{a}$ are MPa and μm, respectively.

It is well-known that for steels manufactured using conventional methods, the endurance limit is lower for rough surfaces. The surface factor which characterises the surface condition depends on tensile strength; For instances, rougher surfaces and materials with higher UTS result in a lower surface factor, hence lower endurance limit [25]. The same concept is adopted in the fatigue model for AM stainless steel parts. The surface factor, $k_{s}$, is a function of the average surface roughness value $R_{a}$ and the UTS. The relationship is described in Figure 2 which is an empirical chart using experimentally obtained $R_{a}$ and UTS values to determine the surface factor. It can be seen that the surface factor is always less than 1 and considers the surface roughness as a source of defect. If a surface has been polished, the surface factor will be very close to 1. However, even if a surface is polished, there might be defects on the surface such as micro-cracks and pits. Defects can exist in the sub-surface and in the bulk of the material in the forms of pores, micro-cracks, and weak grain boundaries as well. Therefore, to overcome the limitations of the surface factor used in the endurance limit approach, a defect factor is introduced to complement the surface factor.

The effects of mean stress, $\sigma_{m}$, can be estimated, using the Goodman approach [28], by adjusting the fatigue stress $\sigma_{a}^{f}$ in Eq. (4) to give:

(5) $$\sigma_{a}^{f}=0.5k_{s}k_{d}\left(1-\sigma_{m}/\sigma_{u}\right)\sigma_{u}\;at\;10^{6}\;cycles$$

where the mean stress, $\sigma_{m}$, is the average of the maximum stress, σ_max_, and the minimum stress, σ_min_, given by:

(6) $$\sigma_{m}=\left(\sigma_{\max}+\sigma_{\min}\right)/2$$

In terms of loading condition, the applied stress amplitude, $\sigma_{a}$, is given by:

(7) $$\sigma_{a}=\left(\sigma_{\max}-\sigma_{\min}\right)/2$$

The stress ratio, R, is the ratio between the minimum stress and the maximum stress given by:

(8) $$R=\sigma_{\min}/\sigma_{\max}$$

Substituting Eq. (8) into Eqs. (6) and (7), the mean stress can be given by:

(9) $$\sigma_{m}=\sigma_{a}\left(1+R\right)/\left(1-R\right)$$

Considering that the applied stress amplitude is equivalent to the fatigue stress amplitude and substituting Eq. (9) into Eq. (5), the fatigue stress amplitude can be given after rearranging by:

(10) $$\sigma_{a}^{f}={0.5k_{s}k_{d}\sigma }_{u}/\left(1+0.5k_{s}k_{d}\left(1+R\right)/\left(1-R\right)\right)at\;10^{6}\;cycles$$

It is more common in industry to express the fatigue stress in terms of a stress range. The fatigue stress amplitude is half the fatigue stress range, i.e., $\sigma_{a}^{f}=\sigma_{r}^{f}/2$. Expressing Eq. (10) in terms of $\sigma_{r}^{f}$, we have:

(11) $$\sigma_{r}^{f}=k_{s}k_{d}\sigma_{u}/\left(1+0.5k_{s}k_{d}\left(1+R\right)/\left(1-R\right)\right)at\;10^{6}\;cycles$$

Eq. (2) can be expressed in terms of fatigue stress range by:

(12) $$\sigma_{r}^{f}=1.8\sigma_{u}at\;10^{3}\;cycles$$

Based on the Basquin fatigue model, the relationship between the fatigue stress range, $\sigma_{r}^{f}$, and the number of cycles to failure, $N_{f}$, for most metals is given by [29]:

(13) $$\sigma_{r}^{f}=A{N_{f}}^{n}$$

where A and n are material constants.

In this study, Eqs. (11) and (12) are used to obtain two points of the S–N curves (stress ranges vs number of cycles). The two points are then used to obtain the material constants, A and n, in Eq. (13) by fitting.

## 3 Application of the model to AM 316L SS

**Figure 3:**
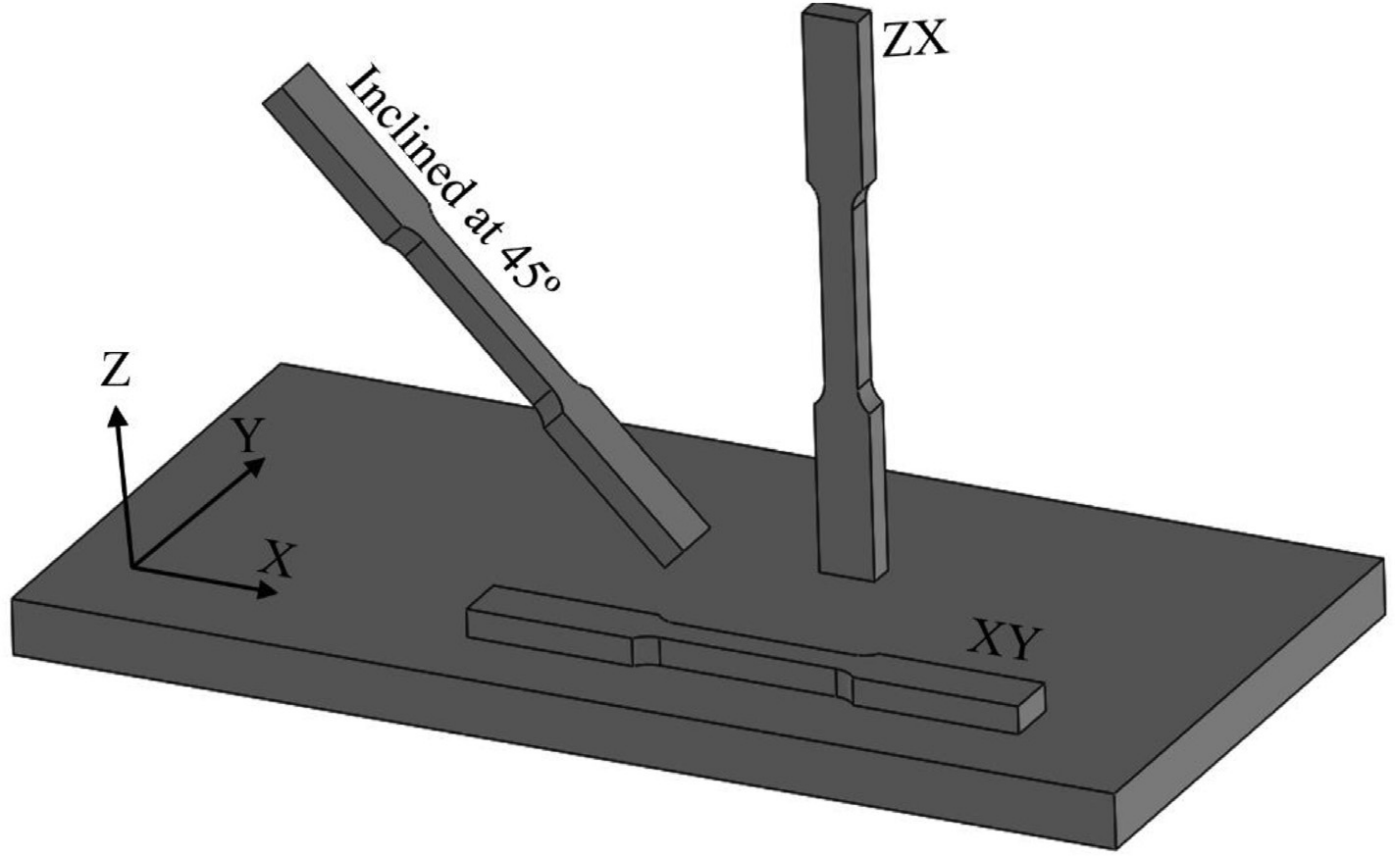
Samples with their longitudinal axis at different orientations with respect to the build plate: horizontal (XY), vertical (ZX) and inclined at 45°.

There are a large number of parameters affecting the quality and mechanical properties of parts produced by metal AM technologies. While there are more than 100 reported process variables for producing AM parts [23], different machines, loading conditions and post-processing variables add to the inhomogeneity of the fabricated parts and in particular their fatigue properties. Fatigue samples are normally printed with their longitudinal axis at different orientations with respect to the build plate: horizontal (XY), vertical (ZX) and inclined at 45°, as shown in Figure 3. Table 1 presents mechanical properties and surface roughness of AM 316L SS parts printed using different machines at XY, ZX and at 45° inclined directions, with various process and post-processing conditions. The tested samples have undergone different load-controlled fatigue loading conditions, namely rotating-bending at R=−1 [30, 31, 32, 33], axial at R=−1 [32, 33, 34, 35, 36, 37, 38, 39, 40] and axial at R=0.1 [22, 36, 41, 42, 43], and at different frequencies.

**Table 1:** Details of AM 316L SS parts printed using different process and post-processing conditions∗.

Printing system	Process condition	Surface roughness, $R_{a}$ (μm)	Failure strain (%)	Yield stress (MPa)	UTS (MPa)	Hardness	Ref.
SLM 250 HL	ZX-AB	13.29	-	-	-	-	[30]
SLM 250 HL	ZX-M (turned)	1.08	-	-	-	-	[30]
SLM 250 HL	ZX-M (vibratory finish)	1.74	-	-	-	-	[30]
SLM 250 HL	ZX-AB-NSR	-	53.7	462	565	-	[40]
SLM 250 HL	ZX-M-SR^1^	-	48.6	443	595	-	[40]
SLM 250 HL	ZX-M-NSR	-	52.5–57.5	-	598–602.4	-	[34]
SLM 250 HL	ZX-M-SR^1^	-	52.5–55.7	-	616.5–619.3	-	[34]
SLM 250 HL	ZX-HIP^2^	-	61.5–67.3	-	583.6–588.4	-	[34]
Renishaw AM250 SLM	XY-AB-NSR	6.7–10.3	54.4–57	542–564	664–665	-	[22]
Renishaw AM250 SLM	XY-AB-SR^3^	6.7–10.3	47.3–49.6	544–556	675–689	-	[22]
Renishaw AM250 SLM	ZX-AB-NSR	18.9–23.5	56.5–65	451–495	587–602	-	[22]
Renishaw AM250 SLM	ZX-AB-SR^3^	18.9–23.5	23.6–32.5	467–495	583–594	-	[22]
Renishaw AM250 SLM	ZX-M or XY-M	0.62–0.70	-	-	-	-	[22]
Renishaw AM250 SLM	ZX-SP-NSR	3.64–4.56	-	-	-	-	[22]
EOS M270 SLM	ZX-M-NSR	0.4	26	530	660	-	[41]
EOS M270 SLM	ZX-Polished-NSR	0.1	-	-	-	-	[41]
EOS M270 SLM	ZX-AB-NSR	10	-	-	-	-	[41]
EOS M270 SLM	Inclined at 45º-AB-SR^4^	5–6	30	473	680	-	[31]
EOS M270 SLM	XY-AB-SR^4^	5–6	28	496	717	-	[31]
SLM	ZX-AB	10.1	-	-	437	-	[42]
ProX®DMP 320 SLM	ZX-AB-NSR	5.9–8.3	45–47	447–459	567–579	-	[38]
ProX®DMP 320 SLM	ZX-AB-SR^5^	5.9–8.3	47–49	444–454	565–575	-	[38]
ProX®DMP 320 SLM	ZX-M	0.44–0.56	-	-	-	-	[38]
ProX®DMP 320 SLM	ZX-M-SA^6^	0.5	56–58	330–346	548–572	-	[39]
ProX®DMP 320 SLM	ZX-M-HIP^7^	0.5	58–60	318–322	553–567	-	[39]
ProX®DMP 320	XY-M	-	38.4–39	572–588	677–679	223–231 (HV30)	[35]
ProX®DMP 320	Inclined at 45º-M	-	35.8–37	579–587	660–664	224–230 (HV30)	[35]
ProX®DMP 320	ZX-M	-	40.6–41.8	513–525	599–601	232–226 (HV30)	[35]
EOS M290	XY-M-NSR	-	40.5–44.9	557–615	716–730		[36]
EOS M290	ZX-M-NSR	-	67.7	500	620		[36]
EOS M290	XY-M-HIP^8^	-	52.2–55	277–289	623–629		[36]
EOS M290	ZX-M-HIP^8^	-	63.8–70.6	272–288	574–590		[36]
EOS M290	XY-M-SR^9^	-	51.9	440	673		[44]
EOS M290	XY-M- SR^10^	-	56.1	420	665		[44]
Renishaw AM250 SLM	ZX-AB-SA^11^	10–14	-	-	-	-	[32, 33, 45]
Renishaw AM250 SLM	ZX-M-SA^11^	1	-	-	-	-	[32, 33, 45]
Trumpf TruPrint 1000	ZX-M	-	33–49	462–496	553–577	-	[37]
EOS M 290	XY-M	2	32	546	654	208 H V	[43]
EOS M 290	XY-AB	12.5	-	-	-	-	[43]
EOS M 290	ZX-AB	6	48	439	553	200 H V	[43]
EOS M 290	ZX-M	2	44	457	569	200 H V	[43]
EOS M 290	ZX-HFMI^12^	2	22	471	647	-	[43]
EOS M100 DMLS	XY-AB	-	35	535	650	-	[46]
EOS M100 DMLS	ZX-AB	-	45	490	590	-	[46]
EOSINT M280 200W, EOSINT M280 400W and EOS M290 400W	XY-AB	-	25–55	470–590	590–690	89 (HRB)	[47]
EOSINT M280 200W, EOSINT M280 400W and EOS M290 400W	ZX-AB	-	30–70	380–560	485–595	89 (HRB)	[47]
EOS M290 DMLS	AB^13^	-	46.7	500	590	-	[48]
EOS M400-4 Laser sintering	XY-AB	-	40	550	650	90 (HRB)	[49]
EOS M400-4 Laser sintering	ZX-AB	-	45	490	590	90 (HRB)	[49]

∗ Note: AB means as-built; M means ‘machined’; SR means stress-relieved; NSR means non-stress-relieved; SP means shot peened; SA means solution annealing.

1 Stress-relieved for 2 h at 650 °C under an argon atmosphere.

2 HIPed under an argon atmosphere for 4 h at pressure of 1000 bar and temperature of 1150 °C.

3 Stress-relieved for 6 h at 470 °C, followed by air cooling.

4 Stress-relieved at 388 °C for 4 h.

5 Stress relieved at 470 °C for 5 h in an argon atmosphere.

6 Solution annealing at 1060 °C for 1 h, followed by argon quenching.

7 HIPed at 1155 °C and applied holding pressure of 100 MPa for a dwell time of 3 h, followed by furnace cooling.

8 HIPed at 1190 °C for 4 h at a pressure of 145 MPa.

9 Stress-relieved at 982 °C for 25 min followed by gas quenching.

10 Stress-relieved at 1093 °C for 25 min followed by gas quenching.

11 Solution annealing at 1038 °C for 1h in vacuum followed by air cooling.

12 High-frequency mechanical impact (HFMI) surface treated with pins of specific speed, travel angle and tilt angle.

13 The numbers are average values determined from samples with horizontal and vertical orientations.

The collected data in Table 1 shows that the average UTS in the vertical direction (584 ± 4.8 MPa) is lower than the one in the horizontal direction (668 ± 28.9 MPa). The surface factors were obtained from Figure 2 using the average UTS values and the average surface roughness ($R_{a}$) values.

For the as-built (AB) conditions, the $R_{a}$ value is in the range of 5–23.5 μm depending on the process variables (see Table 1). In this study, the same surface factor of 0.75 for the horizonal and vertical directions was obtained as provided in Table 2. This is based on Figure 2, where for UTS of 584 MPa (vertical direction) and 668 MPa (horizontal direction), a surface factor of 0.75 represents $R_{a}$ values of approximately 20 μm and 14 μm, respectively. These two values represent the upper range of the reported $R_{a}$ values in Table 1, which is a conservative approach when applied for fatigue assessment of components where the $R_{a}$ value depends on the build orientation [50]. For the machined (M) and shot peened (SP) surfaces, $R_{a}$ values of 1 μm and 4 μm were used, respectively, and the obtained surface factors are provided in Table 2. The defect factor was empirically obtained by generating S–N curves with different defect factor values and comparing the predicted curves with the experimental data collected from the literature conducted at different R ratios and manufacturing conditions. From design point of view, a conservative approach of using S–N data is more common in industry. Therefore, S–N curves obtained from vertically built sample (ZX) are considered more appropriate.

**Table 2:** Surface and defect factors for parts printed at vertical (ZX) or horizontal (XY) directions with different conditions: as-built (AB), machined (M) and shot peened (SP).

Direction	Condition	UTS (MPa)	Surface factor, $k_{s}$	Defect factor, $k_{d}$
ZX	AB	584	0.75	0.6
	M	584	0.95	0.6
	SP	584	0.88	0.7
XY	AB	668	0.75	0.8
	M	668	0.95	0.8

To complement the limitations of the surface factor, which considers only the measured surface roughness as a source of defect, the defect factor that only considers internal defects (in the bulk and not surface of the material) is obtained based on fitting the S–N curves to the data points with the lowest stress range. This approach can capture the worst scenario on the effect of the defects in the tested samples for different manufacturing conditions. For instance, a machined sample improves the surface roughness, but an internal pore could result in a cavity on the surface after machining if the cutting tool intersects a pore. Due to the unpredictability of the type, size and location of defects in AM, this conservative approach in obtaining the defect factor can be considered appropriate in design for AM against fatigue.

**Figure 4:**
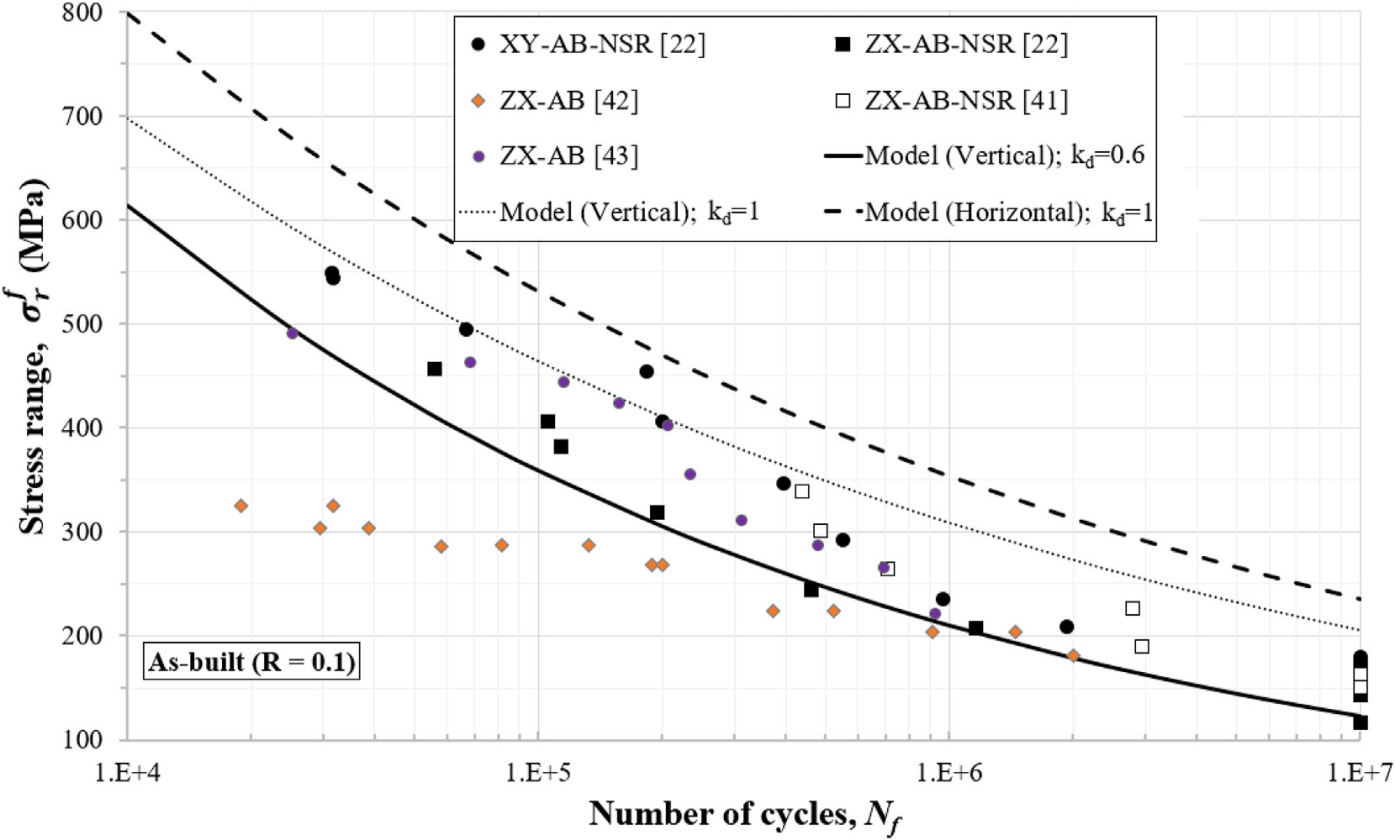
S–N data and predicted curves for as-built samples at R=0.1.

**Figure 5:**
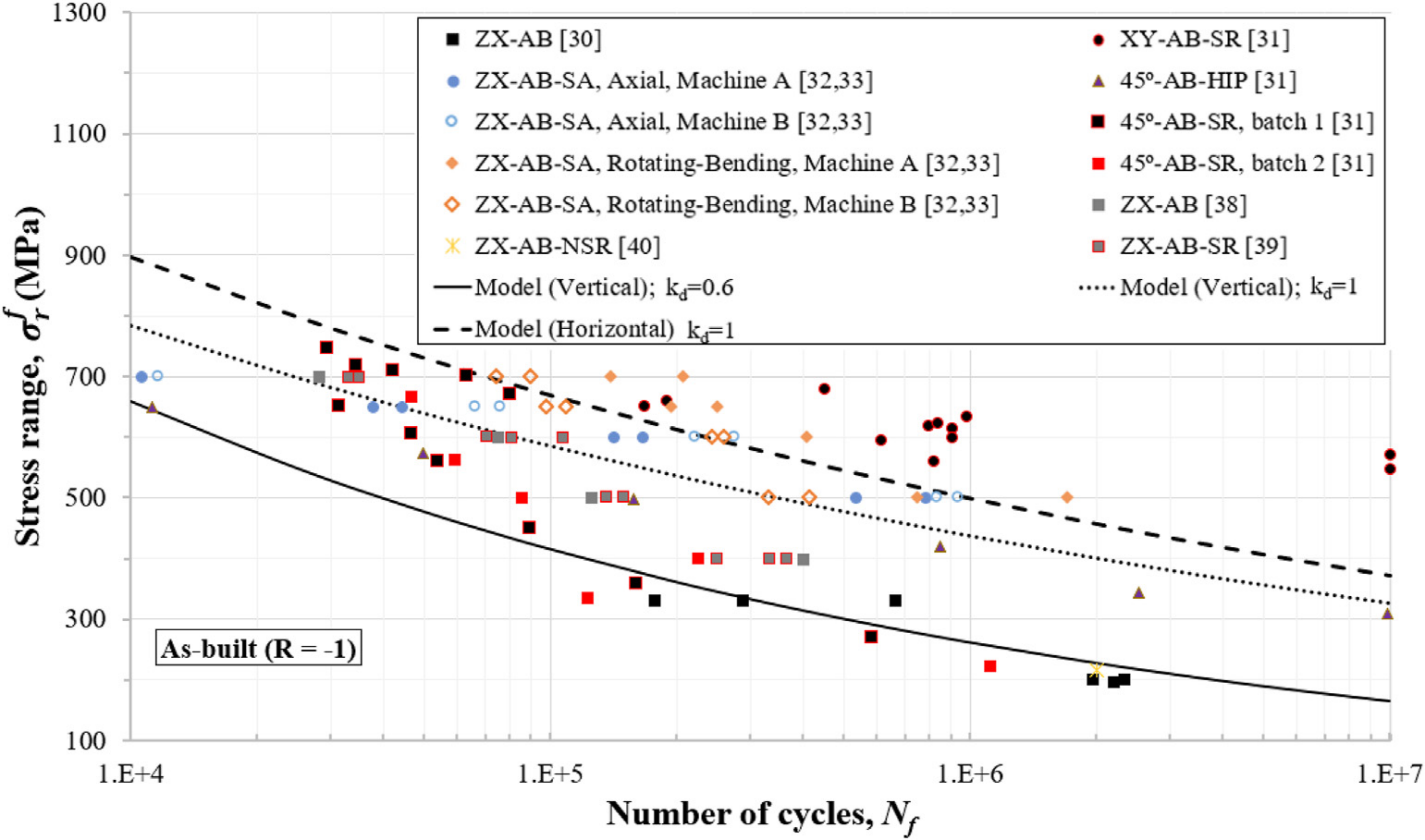
S–N data and predicted curves for as-built samples at R=−1.

**Figure 6:**
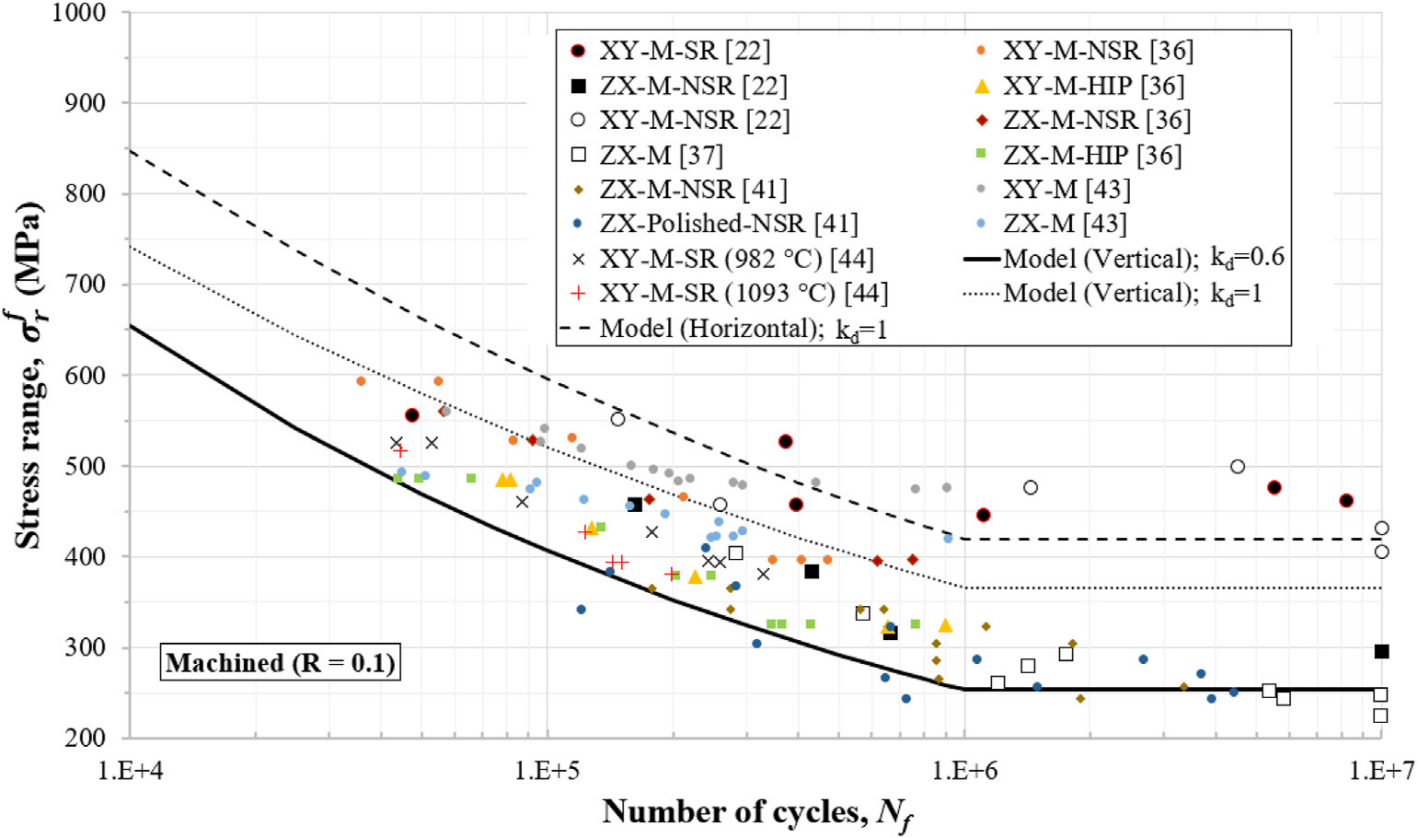
S–N data and predicted curves for machined samples at R=0.1.

**Figure 7:**
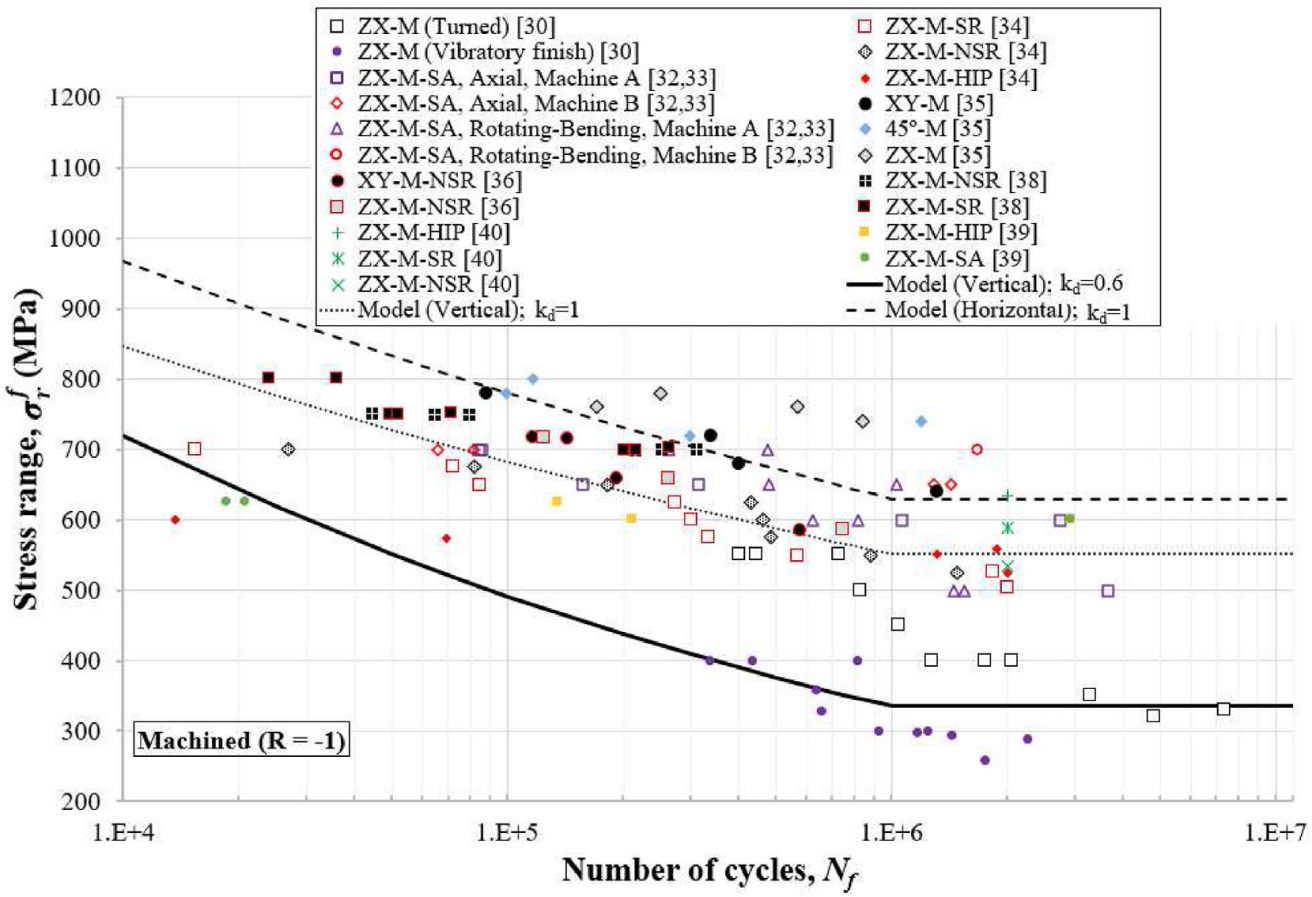
S–N data and predicted curves for machined samples at R=−1.

**Figure 8:**
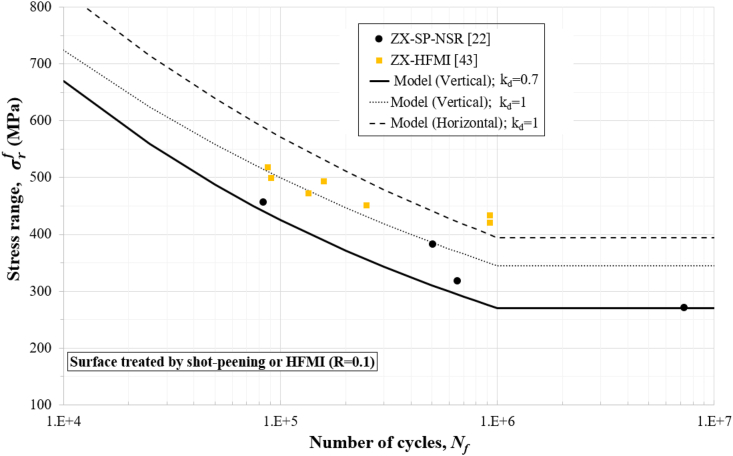
S–N data and predicted curves for shot-peened (SP) or high-frequency mechanical impact (HFMI) surface treated samples at R=0.1.

Figures 4, 5, 6, 7, and 8 show how the obtained defect factors predict the lower bound of the S–N data. A defect factor of 1, i.e., $k_{d}\approx 1$, has a theoretical meaning of no defect, which is impossible in practice. We used a defect factor of 1 in the model to show the theoretical upper bound of the S–N curve. The obtained defect factors are provided in Table 2. The horizontal and 45°-inclined samples have very similar average UTS according to Table 1, hence the surface factor and the defect factor from Table 2 for the horizontal direction can be used for 45°-inclined direction as well. It can be seen that the defect factor for the vertical build orientation is 0.6 while for the horizonal build orientation is 0.8 for both machined and non-machined conditions. The obtained fatigue constants for Eq. (13) after fitting are given in Table 3. The constants for the horizontal (XY) direction can be used for the 45°-inclined direction as well.

**Table 3:** Material constants, A and n, in Eq. (13) related to fatigue loading at different stress ratios, R, for parts printed at vertical (ZX) or horizontal (XY) direction with different conditions: as-built (AB), machined (M) and shot peened (SP).

Direction	Condition	R=0.1		R=−1	
		A	n	A	n
ZX	AB	5246	-0.233	4205	-0.201
	M	4360	-0.206	3320	-0.166
	SP	4112	-0.197	N/A	N/A
XY	AB	4798	-0.2	3607	-0.159
	M	4038	-0.175	2848	-0.125

## 4 Discussion

Figures 4, 5, 6, 7, and 8 include experimental data obtained at different conditions. The first notable observation is that there is a scatter in the data. The scatter of the data is due to several factors:

• First, powder characterisation, process parameters and usage of different machines would affect the fatigue performance.
• Another factor is the size, shape, and location of defects. Large and sharp defects located closer to the surface will generate higher stress intensity leading to local plasticity and propagation of a crack. The defect generation in metal LPBF has a random nature and it is difficult to develop a process that can mitigate defects in full.
• Build orientation is another important factor. In general, the tested samples in the vertical direction exhibit lower fatigue performance.
• Post-heat treatment is another factor where the residual stresses are relieved, and normally, the microstructure and material properties could change due to phase changes or thermo-mechanical mechanisms. For 316L SS, the heat treatment did not show significant effect on the fatigue performance. It has been discussed that annealing used for the purpose of stress-relief does not heavily influence the microstructural characteristics and monotonic properties of AM 316L SS [40]. Furthermore, the AM 316L SS has a high ductility which results in fairly good fatigue and crack growth performance even in the as-built (AB) condition such that post-heat treatment is not needed [34, 40].

There are, however, other alloys such as 17–4 PH SS which, compared to AM 316L SS, are significantly influenced by different post-heat treatment processes in terms of microstructural and monotonic properties, hence considerably affecting their fatigue performance [51, 52]. Therefore, the proposed model in this paper has the potential to be extended for such materials to consider the effects of heat treatment procedures and their interaction with other parameters such as UTS, defects, surface roughness, etc. This is the scope of future works. The current model has the potential to consider other effects such as temperature using Eq. (4). However, there will be a need for experimental data to develop representative temperature correction factors. This is particularly important for AM parts that are designed to operate at low (e. g., space) or elevated temperatures (e. g., power generations). Furthermore, we have investigated cycles up to 10^7^ for AM SS 316L due to limited available data beyond this range. Further data is needed for very high cycle fatigue (above 10^7^ cycles) of AM stainless steels to extend the applicability of the model for longer lives.

Another observation is that the S–N curves for the machined surfaces have a cut-off at 10^6^ cycles (see Figures 6 and 7) which is typical for steel alloys. However, for the as-built conditions (Figures 4 and 5), this phenomenon was not observed, and the fatigue limit continues its reduction between 10^6^ and 10^7^ cycles. Therefore, the cut-off point was not considered in the predicted curves for the as-built specimens. As a reference to design codes for fatigue (e.g., BS 7608), the gradient after 10^7^ cycles is reduced to account for the effect of very high cycle fatigue, instead of using a cut-off value. This approach can be considered conservative when design for very high cycle fatigue applications (above 10^7^ cycles) is needed.

The inputs to the proposed fatigue model are UTS, surface roughness, surface factor and defect factor. The surface factor is a function of the UTS and the surface roughness which are experimentally obtained. As explained in section 3, the model was used to obtain the defect factor by fitting into collected fatigue data from literature at different stress ratios. Three S–N curves were generated using the proposed fatigue model. Two of the S–N curves represent a defect factor of 1, which theoretically means that no defects are present in the material. These two S–N curves were developed using the UTS for vertical and horizontal builds. The S–N curves developed with the higher UTS does capture the upper bound of data points collected from the literature. From the design perspective, the use of such a S–N curve would be considered as a less conservative approach to conduct fatigue analyses. As the vertical build provides the lowest fatigue strength, the UTS for the vertical build was used and a S–N curve with a defect factor of 0.6 was developed. It can be seen that a defect factor of 0.6 represents the lower bound of the data points. The use of UTS for vertical samples and a defect factor of 0.6 would be a pragmatic approach to design industrial AM parts, which have complex geometries and the location, size and type of defects have a random nature.

The limitation of the proposed defect factor is that it does not consider the characteristics of the defect (location, size and shape). One of the main benefits of AM is its ability to produce lightweight components. The use of conservative approaches, such as the use of a defect factor of 0.6, can be considered as a limitation for the lightweight driver in AM. This study gives us the knowledge that defect factors greater than 0.6 can be used, but this would require further inputs to understand the effect of location, size and shape (morphology) of the defect. To remedy the conservativeness of the proposed defect factor, the defect factor will have to be derived as a function of the defect location, size and morphology. Once this relationship is established, X-ray computed tomography (XCT) could be performed to characterise defects and correlate those defects to a defect factor. For instance, large defects with high aspect ratio that are close to the surface would have a defect factor close to 0.6, while small defects with a spherical shape and further from the surface would have a defect factor close to 1. This can enable the life prediction of any AM component by identifying the defect factor using XCT. However, this approach would require further research to identify those relationships.

The current model considers that both the surface factor and the defect factor contribute to the reduction of the fatigue strength. This can be true when the defect is close to the surface and the stress intensities generated at the surface and at the defect are intercorrelated. However, for cases where the defect is at the surface, it is questionable whether the use of the surface factor is needed. The same is also relevant if the defect is further from the surface and the stress intensities at the surface and at the defect are not dependent on each other. In this case, it can be argued that a crack would initiate either at the surface or at the defect, hence only one of the factors should be considered. In this study, it was conservatively assumed (worst-case scenario) that the two factors are intercorrelated due to the random nature of the defect generation in AM.

Stress ranges, $\sigma_{r}^{f}$, are used in Figures 4, 5, 6, 7, and 8 for R=0.1 (tension-tension) and R=−1 (reversed tension-compression). The proposed fatigue model can capture the effect of the R ratio which represents the mean stress effect in real applications. The proposed model with a defect factor of 0.6 can be applied at design stage where either static or dynamic load cases are used. It can be also used for fatigue analyses subject to in-service loading conditions. The key advantage of the proposed model is that it shares common fatigue assessment principles with international standards (e.g., BS7608 and BS8118), which are widely adopted and used in industry. The shared principles include the use of a stress range to estimate the number of cycles by considering the mean stress effect.

In industry, applications of metal AM include the replacement of existing components produced by traditional manufacturing where the load cases for those components may exist. At design stage, the stress levels under the loading conditions can be obtained using the finite element method. In cases where testing equipment exist to mimic the in-service loads at component scale, strain gauges can be employed to measure the strains in different directions and obtain the mean stresses and stress ranges. Knowing the mean stresses and the stress ranges, the proposed model can be used to calculate the number of cycles. For multi-axial loads, cumulative damage theories can be utilised to calculate a critical damage where a crack can initiate (e.g., BS7608). For in-service conditions, strain gauges can be also utilised to measure transient strains. The produced signal can be processed using rainflow algorithms to group the strain ranges and their associate mean stresses [53]. The fatigue model can be employed to calculate the number of cycles and associated damage to estimate the life. The proposed model is practically applicable to industry because it shares some of the fatigue principles adopted into international standards (BS7608, BS8118, Eurocode 3 and Eurocode 9).

As mentioned above, the proposed pragmatic fatigue model is based on industrial practices and has similarities to fatigue codes for design used in industry. The validity of the proposed model and the recommended defect factor of 0.6 for AM 316L SS can be proved by designing parts and monitoring their in-service history or testing designed parts on purposely built test rigs that mimic in-service loads. Similar approach can be applied to other AM materials to identify a safe (conservative) approach for design which might be appropriate for industrial parts where safety is the primary driver.

## 5 Conclusions

The following conclusions were derived from this research paper:

- Fatigue model for development of S–N curves for AM materials based on the endurance limit approach was developed. The model considered the effects of the UTS, mean stress (R ratio), surface finish and defect factor. The defect factor was empirically obtained by fitting the model to collected data from the literature for AM 316L SS. A defect factor of 0.6 was found to capture the lower bounds of the collected S–N data points, which can be used for fatigue assessment at design stage.
- The upper bound of the data was obtained at a defect factor of 1. This indicates that the shape, distance to surface, size of the defect and possibly the number of defects for a given volume could be characterised by the proposed defect factor. For instance, locations with high stress fields at industrial components (“hot spots”) can be scanned with X-ray computed tomography (XCT) to identify the defect and assign a defect factor (i.e., in the range of 0.6–1.0). This method would enable more accurate prediction of fatigue life of AM components.
- The current model has the potential to consider other effects such as temperature for which large experimental data is needed to extract the temperature correction factors. This is particularly important for AM parts that are designed to work at low (e. g., space) or elevated temperatures (e. g., power generations).
- Obtaining the defect factor for other AM materials using the proposed model in this study as well as the relationship between the surface and defects factors will be the focus of future research.
- Associating type, position, size and morphology of defects to the proposed defect factor can be also the focus of future research.

## Declarations

### Author contribution statement

Ahmad Serjouei: Conceived and designed the experiments; Performed the experiments; Analyzed and interpreted the data; Contributed reagents, materials, analysis tools or data; Wrote the paper.

Shukri Afazov: Conceived and designed the experiments; Performed the experiments; Analyzed and interpreted the data; Contributed reagents, materials, analysis tools or data.

### Funding statement

This research did not receive any specific grant from funding agencies in the public, commercial, or not-for-profit sectors.

### Data availability statement

Data included in article/supplementary material/referenced in article.

### Declaration of interests statement

The authors declare no conflict of interest.

### Additional information

No additional information is available for this paper.
